# Identification of Novel Regulatory Small RNAs in *Acinetobacter baumannii*

**DOI:** 10.1371/journal.pone.0093833

**Published:** 2014-04-04

**Authors:** Rajnikant Sharma, Sankalp Arya, Supriya Deepak Patil, Atin Sharma, Pradeep Kumar Jain, Naveen Kumar Navani, Ranjana Pathania

**Affiliations:** 1 Department of Biotechnology, Indian Institute of Technology Roorkee, Roorkee, Uttarakhand, India; 2 National Research Centre on Plant Biotechnology, New Delhi, India; Rush University Medical Center, United States of America

## Abstract

Small RNA (sRNA) molecules are non-coding RNAs that have been implicated in regulation of various cellular processes in living systems, allowing them to adapt to changing environmental conditions. Till date, sRNAs have not been reported in *Acinetobacter baumannii* (*A. baumannii*), which has emerged as a significant multiple drug resistant nosocomial pathogen. In the present study, a combination of bioinformatic and experimental approach was used for identification of novel sRNAs. A total of 31 putative sRNAs were predicted by a combination of two algorithms, sRNAPredict and QRNA. Initially 10 sRNAs were chosen on the basis of lower E- value and three sRNAs (designated as AbsR11, 25 and 28) showed positive signal on Northern blot. These sRNAs are novel in nature as they do not have homologous sequences in other bacterial species. Expression of the three sRNAs was examined in various phases of bacterial growth. Further, the effect of various stress conditions on sRNA gene expression was determined. A detailed investigation revealed differential expression profile of AbsR25 in presence of varying amounts of ethidium bromide (EtBr), suggesting that its expression is influenced by environmental or internal signals such as stress response. A decrease in expression of AbsR25 and concomitant increase in the expression of bioinformatically predicted targets in presence of high EtBr was reverberated by the decrease in target gene expression when AbsR25 was overexpressed. This hints at the negative regulation of target genes by AbsR25. Interestingly, the putative targets include transporter genes and the degree of variation in expression of one of them (A1S_1331) suggests that AbsR25 is involved in regulation of a transporter. This study provides a perspective for future studies of sRNAs and their possible involvement in regulation of antibiotic resistance in bacteria specifically in cryptic *A. baumannii*.

## Introduction

During the last three decades, nosocomial infections caused by multidrug resistant (MDR), Gram-negative bacilli have become a significant health problem. *A. baumannii* is a versatile Gram-negative bacillus that has evolved to be one of the major opportunistic nosocomial pathogens [1], [2]. It causes bloodstream infections, ventilator-associated pneumonia, urinary tract infections, meningitis and wound infections in hospitalized patients; especially the ones with prolonged stays in intensive care units where the host is most vulnerable and the use of antimicrobial agents is highest [3], [4]. *A. baumannii* is a strictly aerobic non-motile bacterium that can adhere to the inanimate surfaces like the hospital equipments and survive on these dry surfaces for long periods of time [5]–[7]. Difficulties in controlling and eradicating the spread of *A. baumannii* have challenged clinicians worldwide. Its intrinsic resistance to a range of antimicrobial compounds, ability to acquire antibiotic resistance genes by horizontal transfer and global spread has led to high mortality in patients infected with this organism [1], [8]–[10].

Resistance of *A. baumannii* to almost every clinically used antibiotic makes treatment of infections extremely difficult [11]. Multidrug resistance in *A. baumannii* is attributed to different mechanisms including degradation, modification, decreased permeability and increased efflux of antibiotic molecules [12]. Multidrug efflux pumps that decrease drug accumulation in the cell, cause severe complications in the treatment of infections caused by *A. baumannii*. Several efflux systems conferring resistance to antimicrobial agents have been described in *A. baumannii* [13]–[18] and many putative efflux pump genes have been identified in its genome sequence [19]. To understand how the bacterium utilizes these efflux pumps, it is important to first explicate the process by which their expression is regulated. The study of the intrinsic regulation of expression of these pumps presents a viable option to elucidate the behaviour and virulence of the pathogen and thus design therapies directed against the pathogen.

Regulation by RNA (Ribonucleic Acid) molecules plays a significant role in amending gene expression in all living systems including bacteria. The largest and most extensively studied bacterial regulatory RNAs are non-coding RNAs commonly referred as small RNA (sRNA) due to their small size (50–500 nucleotides) [20], [21]. sRNAs control the expression of genes by base pairing to their cognate target mRNA and modulating the translational activity and/or the stability of mRNA. sRNAs are significant to a pathogen as they regulate virulence [21]–[26] and susceptibility to different stress conditions [27]–[30]. Initially, sRNAs were identified in *Escherichia coli,* however, with increase in availability of bacterial genome sequence, many sRNAs have been identified in pathogenic bacteria [31] like *Mycobacterium tuberculosis* [32], *Salmonella* spp. [33], [34], *Listeria monocytogenes* [35], *Pseudomonas* spp. [36]–[39], *Vibrio* spp. [40], [41] and *Staphylococcus* spp. [42]–[45]. A number of these sRNAs have been implicated in the regulation of genes significant for bacterial drug resistance [46]–[49].

Although it is widely accepted that sRNAs are encoded by most if not all bacteria, genome-wide annotations for sRNA-encoding genes have not been conducted in the clinically significant pathogen, *A. baumannii*. Due to the importance of sRNAs as regulators, it is likely that they may have vital regulatory roles in drug resistance of *A. baumannii*. In the study reported here, a systematic search for sRNAs focussing on intergenic regions has been undertaken in *A. baumannii*. This search involved computational approaches followed by verification by Northern blotting leading to the novel sRNAs that we refer to as AbsR1 to 31 (for *Acinetobacter baumannii* small RNA 1 to 31). Three novel small RNAs were identified and verified in this study. Out of the three novel small RNAs identified, targets of one of the sRNA, AbsR25 were predicted by *in silico* target analysis in combination with qRT-PCR (quantitative real time PCR). This study demonstrates for the first time the presence of sRNAs in the nosocomial pathogen *A. baumannii* and the expression of AbsR25 in different stress conditions.

## Materials and Methods

### Sequence data and databases

ORF databases, tRNA and rRNA coordinates were obtained from the national center for biotechnology information (NCBI) ftp database (ftp://ftp.ncbi.nih.gov/genomes/Bacteria/). Locus and product names were already mentioned in the database obtained from NCBI. Genomes of all the bacteria used in the present study were downloaded from NCBI, in FASTA format for basic local alignment search tool (BLAST) analysis.

### Prediction of sRNA loci using bioinformatic tools

Candidate sRNAs in *Acinetobacter baumannii* ATCC17978 were predicted using the sRNAPredict program [50] and other bioinformatic tools listed below:

#### AB-BLAST

This is a new version of BLAST developed by the Advanced Biocomputing Company based on the previous WU-BLAST developed by Warren R. Gish for Washington University. For AB-BLAST against *Pseudomonas aeruginosa*, *Burkholderia multivorans* and *Stenotrophomonas maltophilia,* the parameters were set as follows: word length (W) 7, match/mismatch scores (M/N) 5/-4, penalty score for extending a gap (R) 10 and penalty for a gap of length one (Q) 10, and maximum E-value 1e-05.

These parameters correspond to default parameters of WU-BLAST 2.0, the software used in the study of *P. aeruginosa* sRNAs by Jonathan Livny *et al* [50]. The same parameters were used for BLAST against the genomes of *Acinetobacter* spp. (*Acinetobacter baylyi* ADP1 and *Acinetobacter* DR1) in a parallel study.

#### QRNA

It is a probabilistic model for predicting conserved secondary structure and its best probable fit. This program has three models, one for each of RNA, coding region and something else (for others). So, given a pairwise sequence alignment, it calculates Bayesian probability whether the sequence should correspond to an RNA, a coding region or something else. This is given in the output as ‘winner  =  <RNA/COD/OTH>’ for RNA, coding region or other respectively [51], [52]. For QRNA script ‘blastn2qrnadepth.pl’ the parameter for max e-value was set to 1e-05. Further, for the QRNA program, the parameter for window span was set to 150 for database of *P. aeruginosa*, *B. multivorans* and *S. maltophilia* and 100 for database of two *Acinetobacter* spp. genomes and the slide size was 50 in both cases. Also, in the case of two *Acinetobacter* spp., the minimum length was set to 50 base pairs.

#### RNAMotif

This program takes a fasta formatted sequence file and a descriptor file, which gives the program a description of the key features of the structure to be looked for in the sequences, as input. For our purpose, the descriptor file gave the description of the key features of a rho-independent terminator [53]. This descriptor file was provided by D.J. Ecker, the creator of the program, to Jonathan Livny for his study of sRNAs of *P. aeruginosa* [50] and *Vibrio cholerae*. This file was available with the sRNAPredict3 program as it can take input directly from RNAMotif if this file is used as descriptor file.

#### TransTermHP

It is a web-based software for predicting rho-independent terminators in a genome [54]. For our purpose, we kept the default settings except for the confidence level of the predicted terminators which was set to 96%.

#### IGRExtract–

This program takes the fasta format file of the whole genome as an input along with the Genbank file and ptt file for protein coding regions [55]. From these, it calculates the coordinates of inter genomic regions and extracts the sequences from the fasta file.

#### sRNAPredict3

It is a C^++^ program that predicts sRNA using a coordinate based algorithm [55]. This algorithm requires the results of all the programs listed above to identify sRNA by searching for co-localization of genetic features predicted by these programs. These features have been generally known to be associated with genes encoding for sRNA such as rho-independent terminators (from RNAMotif and TransTermHP), secondary structure conservation (from QRNA), etc. The rRNA, tRNA and ORF loci were excluded by the sRNAPredict from its output, which were downloaded from NCBI database.

### Bacterial strains and growth conditions

*A. baumannii* MTCC1425 (ATCC15308) and *E. coli* DH5α were the bacterial strains used for this study. The strains were grown on nutrient agar (Himedia, India) and Luria Bertani (LB) agar (Merck, Germany) medium respectively. For liquid cultures, *A. baumannii* was grown in nutrient broth at 30°C, 200 rpm and *E. coli* was grown in LB broth at 37°C under agitation (200 rpm). pTZ57R/T cloning vector (Fermentas, USA) was used for cloning RACE PCR products. Whenever needed, ampicillin was added to the LB broth at a final concentration of 100 μg/ml.

### Plasmid and strain construction

Standard methods were used for plasmid isolation, DNA purification, restriction endonuclease cleavage, ligation and transformation [56]. Over-expression of AbsR25 was achieved under the control of arabinose inducible promoter P_BAD_. pPROBE-NT [57], a broad host range promoter less plasmid, was used for cloning the promoter (P_BAD_) and AbsR25. The plasmid was modified for replication in *A. baumannii* by cloning 1337 bp *ori* region from pWH1266 [58], a *Acinetobacter* genus specific plasmid, in *EcoR*I restriction site of pPROBE-NT. P_BAD_ promoter containing araC, required for tight regulation, was amplified form pBAD30 plasmid and AbsR25 was amplified from purified chromosomal DNA of *A. baumannii* MTCC 1425. P_BAD_ promoter was cloned within *Sac*I and *Xba*I enzyme sites of the plasmid and AbsR25 was cloned downstream of the promoter within *Sal*I and *Hind*III sites. After each ligation the ligated product was transformed into *E. coli* DH5α cells and grown on LB plates containing 40 μg/ml kanamycin. Plasmids were recovered from the positive transformants and confirmed by digestion with respective enzymes. These cloning steps resulted in plasmid pPROBE-P_BAD_-*absR25* expressing *absR25* under the control of arabinose inducible promoter. Primer sequences used are listed in Table 1. Furthermore the plasmid was sequenced and transformed into *A. baumannii* MTCC 1425 cells and selected by growth in LB agar plats containing 40 μg/ml kanamycin. 2% L-arabinose was used for induction of the promoter for 5 hrs.

**Table 1:** Oligonucleotides used in this study.

Oligonucleotide	Sequence	Description
AbsR11_5′_RACE	ACA ACC TCG GTG AAG AGT CCC	For RACE mapping
AbsR11_3′_RACE	GAGAAGTCTAATTTTTAAGACTAGCG	For RACE mapping
AbsR25_5′_RACE	GCTTTTTTAACTTTAGCATCG	For RACE mapping
AbsR25_3′_RACE	TTTAAGTTCCTTTTAAATCATGTG	For RACE mapping
AbsR28_5′_RACE	CGGACAAGTGAATTAAATTTGTAC	For RACE mapping
AbsR28_3′_RACE	CAAGGTAAAACACAGTAACGA	For RACE mapping
ABFP3251	GCCACATAGTCCCCAAAAGA	primer for qRT-PCR
ABRP3251	TTCTTGCAGTTTGTGGAACG	primer for qRT-PCR
ABFP1791	TAGAATGTCGCGAAGACACG	primer for qRT-PCR
ABRP1791	TGCAGAAGTACCGAGCAATG	primer for qRT-PCR
ABFP2269	GCAATTAGCTGAAAGTCGGC	primer for qRT-PCR
ABRP2269	GTGGTGATAGTGCCATGTCG	primer for qRT-PCR
ABFP2584	CGCCAACCAAAGAAGTGAAT	primer for qRT-PCR
ABRP2584	TTCAGGGTATTGGGGTTTCA	primer for qRT-PCR
ABFP2660	ATAGATGTCATTACGGCGGC	primer for qRT-PCR
ABRP2660	ATTGGTTTAGCGTGGACAGG	primer for qRT-PCR
ABFP1331	TGCTGAGAACCAGAATGCAC	primer for qRT-PCR
ABRP1331	AGGTTTTGCTTTTGGTGGTG	primer for qRT-PCR
AB16S_rRNAFP	CAGCTCGTGTCGTGAGATGT	primer for qRT-PCR
AB16S_rRNARP	CGTAAGGGCCATGATGACTT	primer for qRT-PCR
FPAB0229	ACGAATATAGCCAAGTGCCG	primer for qRT-PCR
RPAB0229	CTTGCGTAGCCCAAGAAGTC	primer for qRT-PCR
FPAB1505	TCATGGCCACAACACCTTTA	primer for qRT-PCR
RPAB1505	AAGTCAAAGCACCGGACATC	primer for qRT-PCR
FPAB3401	TGCAACCCCCGAAATAAATA	primer for qRT-PCR
RPAB3401	CTCGGTCCATCGATCGTTAT	primer for qRT-PCR
FPAB3342	AAGAAGAACAAACGCGTGCT	primer for qRT-PCR
RPAB3342	TCAAAGCCCACAAGATACCC	primer for qRT-PCR
FPAB1627	TCTTCTAGACCGTGCTTGGG	primer for qRT-PCR
RPAB1627	TGATTTTCTGAAGCGTGGTT	primer for qRT-PCR
FPpWHori	TAGTGAATTCGATCGTAGAAATATCTATG	Primer for Ori cloning
RPpWHori	TAATGAATTCGGATTTTAACATTTTGCGTTG	Primer for Ori cloning
AbsR25FP	TTTTGTCGACCATAGGTGTTAAGTTTTAAGTTCC	Primer for AbsR25
AbsR25RP	TGCTAAGCTTGGAATAATAATACAAAGAAAAAAGCCTAC	Primer for AbsR25
pBADFP	ATATGAGCTCATGCATAATGTGCCTGTCAAATGG	Primer for promoter
pBADRP	ATATTCTAGATAGCCCAAAAAAACGGGTATGGAG	Primer for promoter
AbsR11_probe	ATGCCCTACAACGTAGCGGTGTCACATCATTGAGAAGTCTAATTTTTAAGACTAGCGTTCTCAATCATGCGACCTGTCTC	Sequence for synthesis of riboprobe for AbsR11 sRNA detection
AbsR25_probe	TCATTCAAAGAAAAACAAACATAGGTGTTAAGTTTTAAGTTCCTTTTAAATCATGTGTAGGACCGAGCTACCTGTCTC	Sequence for synthesis of riboprobe for AbsR25 sRNA detection
AbsR28_probe	AAAAAGGAGGACATCATGCCAACACTACAAGGTAAAACACAGTAACGATTTGGCACGATGTCAGCTCACGCCTGTCTC	Sequence for synthesis of riboprobe for AbsR28 sRNA detection

### Total RNA isolation and Northern blot analysis

For total RNA isolation, a single colony of *A. baumannii* MTCC1425 was grown overnight in a nutrient broth medium at 30°C. The culture was diluted to 1∶100 times and grown at 30°C in 250 ml flasks with shaking at 200 rpm. Cells were harvested from culture grown to an optical density at 600 nm (OD_600_) of 0.4 (lag phase), 1 (early exponential phase), 1.6 (exponential phase) and 3.2 (stationary phase). Temperature, sodium chloride (NaCl) and ethidium bromide (EtBr) stresses were applied to the cells when the *A. baumannii* culture reached an OD_600 nm_ of 0.4. For the temperature stress, the culture was incubated at different temperatures for 30 min and for NaCl stress, cultures were incubated with different concentrations of NaCl for 30 min. EtBr stress was provided by incubating culture with different concentrations of EtBr. Total RNA was isolated with TRI reagent (MRC, Cincinnati, Ohio, USA) according to manufacturers' instructions. The removal of any contaminating DNA was carried out by treating the RNA sample with DNase I (Fermentas, USA).

Twenty microgram of total RNA was separated on 10% denaturing polyacrylamide gel containing 7 M urea and 1X TBE (89 mM Tris-base, 89 mM boric acid and 2 mM ETDA). A low molecular weight RNA ladder was also separated on the same polyacrylamide gel. Following electrophoresis in 1X TBE, the total RNA and ladder were transferred from the gel to a positively charged nylon membrane (Ambion, Austin, TX, USA) in transfer buffer (240 μM glycine, 31.16 μM Tris base, 2.7 M methanol) via electroblotting. The membrane was baked in oven for 1 hrs at 80°C and was pre-hybridized in ULTRAhyb buffer (Ambion, Austin, TX, USA) for two hrs at 42°C in a shaker. The riboprobes (for validation of sRNA) were synthesized by *in vitro* transcription and had complementarity with candidate sRNA. The *mir*Vana probe construction kit (Ambion, Austin, TX, USA) was used for *in vitro* transcription. Biotin labelling of riboprobes was performed with Bright Star psoralen biotin kit (Ambion, Austin, TX, USA) according to the manufacturer's instructions. On an average, the riboprobes were 80 nucleotides in length. The biotin labelled riboprobes were incubated with the pre-hybridized membranes at 42°C in a shaker overnight. The membranes were washed first with low stringency wash buffer (Ambion, Austin, TX, USA) at room temperature for two times, 5 minutes each and then with high stringency wash buffer (Ambion, Austin, TX, USA) for two times, 10 minutes each at 42°C according to manufacturer's instructions. Following washes, the blots were developed (Bright Star Bio-detection kit, Ambion, Austin, TX, USA) and exposed to X-ray film for their visualization.

### 5′ and 3′ rapid amplification of cDNA ends (5′and 3′RACE)

5′ RACE was carried out on DNA free total RNA isolated previously, using the First Choice RLM-RACE kit (Ambion, Austin, TX, USA) according to the manufacturer's instructions with slight modifications. Calf Intestinal Phosphatase (CIP) treatment at the beginning was omitted, because prokaryotic RNA was used and a control without Tobacco Acid Pyrophosphatase (TAP) treatment was included. The AbsR specific primers (Table 1) were used in PCR steps of 5′RACE protocol and Hot star DNA polymerase (Qiagen, USA) was employed for amplification of the sRNA genes. The 5′ RACE PCR product was gel purified and cloned into pTZ57R/T cloning vector (Fermentas, USA). The potential clones were selected, the plasmid DNA was isolated and sequenced for identification of transcription start site.

For 3′ RACE, the total RNA was polyadenylated using Poly (A) Polymerase enzyme (New England Biolabs, USA) according to the manufacturer's instructions. Polyadenylated RNA was then reversed transcribed and PCR amplified by using First choice RLM RACE kit (Ambion, Austin, TX, USA). The PCR product was cloned into pTZ57R/T cloning vector (Fermentas, USA) and sequenced for identification of the transcription termination site.

### Target prediction of AbsR25 sRNA

In order to predict target mRNA genes for the identified AbsR25, sRNATarget (http://ccb.bmi.ac.cn/srnatarget/prediction.php) [59] and IntaRNA (http://rna.informatik.uni-freiburg.de:8080/IntaRNA.jsp) [60] servers were used. Target genes with the score 1 in sRNAtarget were used for comparison analysis with IntaRNA output. For sRNAtarget, the default parameters were used. The default parameters for IntaRNA were as follows: Exact number of base pairs in seed: 7, folding window size- mRNA: 140, maximum base distance- mRNA: 70, maximum energy of interaction: 0 kcal/ml.

### Quantitative Real Time-Polymerase Chain Reaction (qRT-PCR)

qRT-PCR was performed for detection of expression of bioinformatically predicted putative targets of AbsR25 sRNA. For sample preparation, a single colony of *A. baumannii* was grown overnight in nutrient broth at 30°C. This culture was diluted 100 times in 10 ml nutrient broth medium and grown till OD_600_ reached 0.4. When OD_600_ reached the desired point, the cultures were stressed with different concentrations (0, 64, 128, 192 and 256 μg/ml) of EtBr and grown for 18 hrs at 30°C with shaking. After incubation, cells were pelleted and total RNA was isolated from each sample with TRI reagent (MRC, Cincinnati, Ohio, USA) according to manufacturer's instructions, followed by DNase I (Fermentas, USA) treatment for removal of contaminating genomic DNA. Reverse transcription was performed for each stressed sample with Moloney Murine leukemia Virus reverse transcriptase (Promega, Madison, USA) and random hexamer primer. Negative control reactions were performed using equal concentrations of RNA but without Reverse transcriptase. Primers for Real Time PCR were designed with Primer3 software and are listed in Table 1. 16S rRNA was used as internal control for normalization of target mRNA gene expression. All cDNA aliquots for each stress condition were used in equal concentration in a qRT-PCR reaction mix containing 12.5 μl LightCycler 480 SYBR Green I master mix (Roche diagnostics, Germany), 0.2 μM each primer (forward and reverse) and reaction volume was made up to 25 μl with nuclease free water. The PCR was run on a Smart Cycler (Cepheid, Sunnyvale, CA) with an initial denaturation for 5 min at 95°C and a subsequent run of 35 cycles, each comprising 15 sec at 95°C, 30 sec at 50°C and 30 sec at 72°C. The C_T_ (threshold cycle) values from all RT-PCR reactions in triplicate were analysed to detect target mRNA genes expression.

AbsR25 was over-expressed under the control of arabinose inducible promoter and qRT-PCR was used to analyze the effect of over-expression of *absR25* on target genes. For sample preparation, a single colony of *A. baumannii* harbouring recombinant plasmid, pPROBE-P_BAD_-*absR25*, was grown overnight in nutrient broth at 30°C. This overnight culture was diluted 100 times in 10 ml nutrient broth medium and grown till OD_600_ reached 0.6. When OD_600_ reached the desired point, the culture was induced with 2% L-arabinose and grown for 5 hrs at 30°C with shaking. After incubation, cells were pelleted and total RNA was isolated from each sample with TRI reagent (MRC, Cincinnati, Ohio, USA) according to manufacturer's instructions, followed by DNase I (Fermentas, USA) treatment for removal of contaminating genomic DNA. All other steps of qRT-PCR were performed as mentioned earlier.

## Results

### Prediction of putative sRNAs using bioinformatic approach

There are several experimental strategies used for prediction of sRNA that are based on shotgun cloning and microarray methods but computational predictions and validation with Northern blotting have been popularly used for identification of sRNAs in many bacteria [61]. The basis for genome wide screening of small RNAs is their sequence or structural conservation among closely related species. The algorithms that are based on sequence conservation like sRNAPredict [50], [55] and GMMI [62] first search the IGRs (intergenic regions) for transcriptional signal sequences like promoters and terminators and then predict nucleotide conservation. Since the sequence homology is typically based on the primary structure of RNA, so the precision of this method may not be sufficient [63]. Hence some algorithms like QRNA are required [64] that search for phylogenetic conservation of secondary structure. In this study, we employed a combination of sRNAPredict and QRNA algorithms to increase the prediction accuracy.

When this study for sRNA prediction was initiated, among *Acinetobacter* species, genome of only *Acinetobacter baumannii* ATCC 17978 was sequenced. Therefore closely related bacterial genomes were taken for comparative analysis on the basis of *16S rRNA* gene sequences. In the meantime, other *Acinetobacter* species genome sequences also became available. There were, thus, two parallel studies for sRNA prediction by using the same methodology (using QRNA and sRNAPredict program), one with closely related bacterial genomes and another with *Acinetobacter* spp. (*Acinetobacter baylyi* ADP1 and *Acinetobacter* sp. DR1). Overlapping results from the two studies increased the level of confidence in the prediction of putative sRNAs and narrowed down the range of putative sRNAs to be verified.

**Figure 1:**
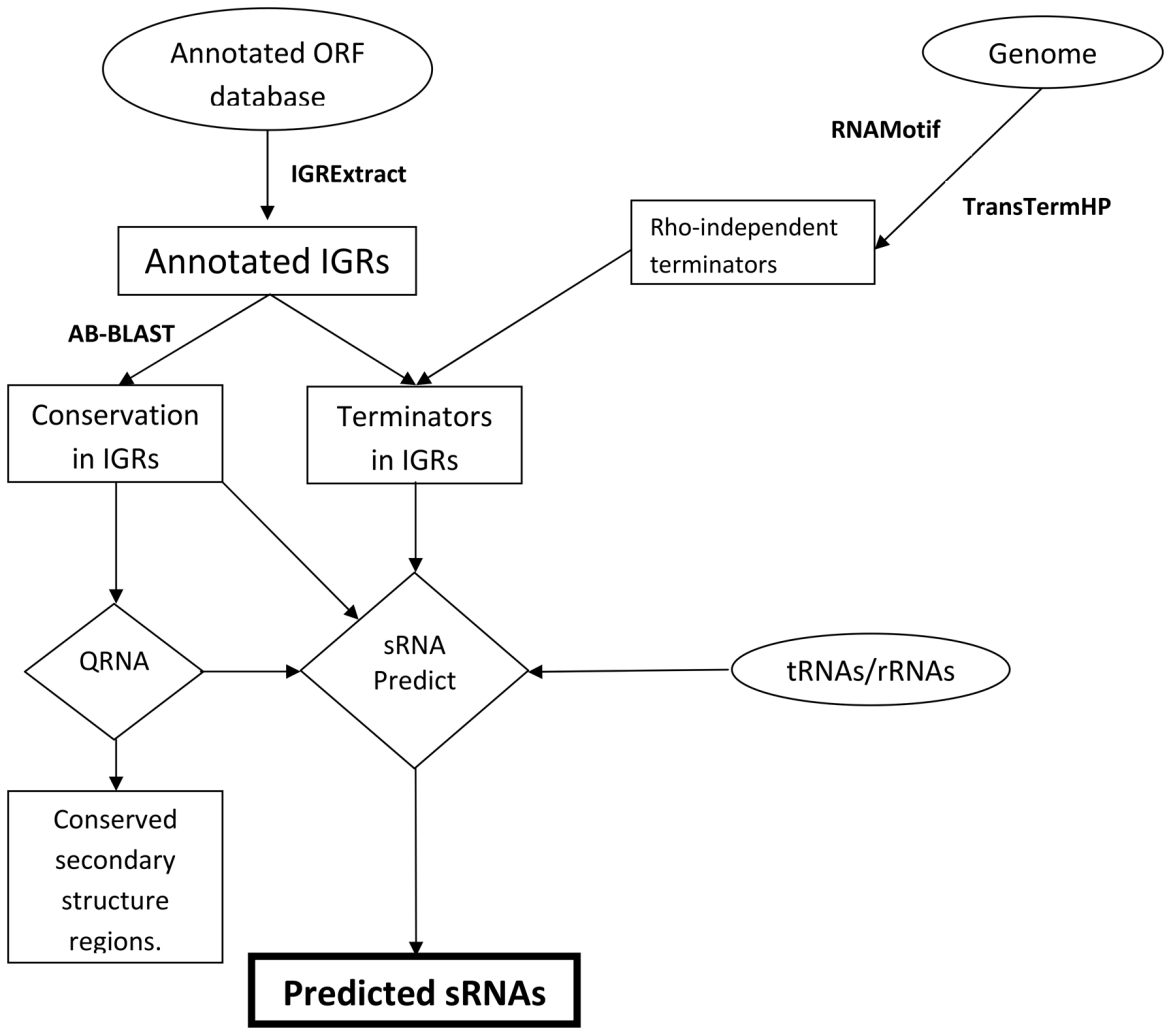
Schematic representation of bioinformatic approach used for small RNA species prediction in *Acinetobacter baumannii* ATCC 17978. The first step was to determine the inter-genic regions (IGRs) from the annotated ORFs (Open Reading frames) and then running them through BLAST against genomes of related species. The conserved sequences were subjected to QRNA program wherein conserved secondary structure regions among the pairwise aligned sequences were determined.

In the study reported here, several steps were employed for prediction of sRNAs of *A. baumannii* (Figure 1). The phylogenetic relationship of *A. baumannii* with other bacteria was determined by aligning their *16S rRNA* gene sequences. The alignment showed that *A. baumannii* was closest to *P. aeruginosa*, *S. maltophilia* and *B. multivorans* (data not given). The *16S rRNA* gene sequences of *A. baumannii* and *P. aeruginosa* showed 88% similarity (100% query coverage) whereas the rest of the sequences showed 85% similarity (100% query coverage) with *A. baumannii* ATCC 17978 genome. Initially, comparative genomics was started with these genomes. Similar comparative genomics for sRNA prediction was carried out again when genome sequences of *A. baylyi* ADP1 and *Acinetobacter* sp. DR1 became available.

IGRExtract was used for extracting intergenic regions (IGRs) from respective genomes for producing IGR databases. These databases were used as subject sequences in BLAST search and converted into coordinates of conserved sequences in the chromosome by sRNAPredict program. IGRs of *A. baumannii* were extracted and used as query for BLAST search against databases of *P. aeruginosa*, *B. multivorans* and *S. maltophilia* genomes.

BLAST alignments were used as input for QRNA. QRNA classified a total of 89 candidate structural RNA loci in *A. baumannii* (Table S1). Thus, there are 89 sequences in IGR of *A. baumannii* with secondary structure conserved across all the three genomes. For further analysis through sRNAPredict3, RNAMotif and TransTermHP were used for predicting rho-independent terminators, and BLAST was done again with the IGR sequences of *A. baumannii* as database. sRNAPredict gave 164 coordinate pairs (Table S2) of which only seven lie in or have regions predicted by QRNA to have conserved secondary structure.

These results were used later for comparison with results from further analysis, done using other *Acinetobacters* spp. namely *Acinetobacter baylyi* ADP1 and *Acinetobacter* spp. DR1. The genome sequences of these were taken as a database for BLAST against *A. baumannii* IGR sequences and provided too many results with the parameters used previously as they were calibrated for 65–100% similar sequences; so default parameters were used which provide 95–100% similar sequences. Running the result through QRNA provided 806 coordinate pairs for conserved secondary structure (Table S3). For sRNAPredict, BLAST was done again with IGR sequences as database and the genomes (*A. baylyi* ADP1 and *Acinetobacter* spp. DR1) as a query. The result of sRNAPredict was 748 putative sRNAs (Table S4) of which 143 were predicted by QRNA as having conserved secondary structure.

Comparing these 748 with the 164 obtained before gave 149 coordinate pairs with either the same start coordinate or same end coordinate or both. Of these 149, only 31 are such which were predicted by QRNA as having conserved secondary structure (Table 2). On the basis of these programs (sRNAPredict and QRNA), 31 putative sRNA genes were predicted in the genome of *A. baumannii* ATCC 17978 (Table 2). These 31 putative sRNAs were predicted based on their location in the intergenic region, conserved secondary structure and termination by rho independent terminator. The genes encoding the sRNA are denoted as *absR* (1 to 31) for *Acinetobacter baumannii* small RNA.

**Table 2:** List of putative 31 sRNAs and their features.

sRNA	Up ORF name	Up ORF dir.	dist Up ORF	sRNA start	sRNA end	direction sRNA	length sRNA	dist Dn ORF	Dn ORF dir.	Dn ORF name	BLAST expect	Term Type
AbsR-1	50S ribosomal protein L1	>>>	0	304073	304175	>>>	102	390	>>>	50S ribosomal protein	2.00E-155	R
AbsR-2	pyrroline-5-carboxylate reductase	>>>	1518	1439995	1440255	>>>	260	295	>>>	phage putative head morphogenesis protein	1.40E-120	R
AbsR-3	amino acid ABC transporter permease	<<<	0	2665457	2665731	>>>	274	11	<<<	lysine/arginine/ornithine ABC transporter periplasmic-binding protein	5.50E-120	R
AbsR-4	lysine-specific permease	>>>	36	3285829	3285889	>>>	60	280	>>>	hypothetical protein	7.50E-15	R
AbsR-5	hypothetical protein	<<<	0	3765974	3766394	>>>	420	45	<<<	putative pirin-like protein	5.40E-157	R
AbsR-6	uracil transport protein	>>>	8	3965175	3965381	<<<	206	113	>>>	putative transcriptional regulator	4.50E-53	R
AbsR-7	glucosamine—fructose-6-phosphate aminotransferase	>>>	608	3904650	3904740	<<<	90	2343	>>>	hypothetical protein	5.80E-200	R
AbsR-8	hypothetical protein	<<<	392	3766366	3766439	<<<	73	0	<<<	putative pirin-like protein	5.40E-157	R
AbsR-9	putative sensory transduction histidine kinase	<<<	1559	3362935	3363158	<<<	223	161	>>>	hypothetical protein	8.30E-293	R
AbsR-10	transcriptional factor	>>>	17	2543367	2543541	<<<	174	195	<<<	tRNA uridine 5-carboxymethylaminomethyl modification enzyme GidA	5.40E-62	R
AbsR-11	hypothetical protein	>>>	35	2529183	2529534	<<<	351	0	>>>	cytochrome o ubiquinol oxidase subunit II	1.80E-202	R
AbsR-12	hypothetical protein	>>>	1307	1956579	1956712	<<<	133	232	>>>	putative methyltransferase	4.40E-150	R
AbsR-13	OmpA/MotB	>>>	33	1398936	1399168	<<<	232	75	>>>	hypothetical protein	9.60E-34	R
AbsR-14	hypothetical protein	<<<	1151	940263	940781	<<<	518	12	<<<	putative transcriptional regulator	1.90E-16	R
AbsR-15	putative transposase	>>>	30	798366	798883	<<<	517	16	<<<	dihydropteroate synthase	4.00E-240	R
AbsR-16	acyl-CoA synthetase	<<<	156	639378	639744	<<<	366	401	>>>	threonyl-tRNA synthetase	3.60E-233	R
AbsR-17	F0F1 ATP synthase subunit epsilon	>>>	20	178139	178326	<<<	187	0	>>>	hypothetical protein	1.10E-106	R
AbsR-18	putative phosphatase	<<<	403	501115	501198	<<<	83	2	<<<	putative alkaline phosphatase	4.70E-130	T
AbsR-19	integration host factor subunit alpha	>>>	2356	654179	654249	<<<	70	0	<<<	hypothetical protein	0	T
AbsR-20	Transposase	>>>	312	800944	801007	<<<	63	1	<<<	rRNA	1.20E-42	T
AbsR-21	putative homocysteine S-methyltransferase family protein	<<<	862	1071839	1071925	<<<	86	3	<<<	malate:quinone oxidoreductase	6.40E-221	T
AbsR-22	major facilitator superfamily fosmidomycin/multidrug transport protein	>>>	1084	1115025	1115571	>>>	546	26	<<<	mutarotase precursor	6.40E-20	B
AbsR-23	OmpA/MotB	>>>	10	1398913	1399168	<<<	255	75	>>>	hypothetical protein	9.60E-34	T
AbsR-24	putative hemolysin	>>>	0	1548816	1548878	>>>	62	910	>>>	dihydrodipicolinate synthetase	0	B
AbsR-25	putative hemolysin	>>>	0	1548816	1549149	>>>	333	639	>>>	dihydrodipicolinate synthetase	0	B
AbsR-26	hypothetical protein	>>>	1289	1956561	1956712	<<<	151	232	>>>	putative methyltransferase	4.40E-150	T
AbsR-27	hypothetical protein	>>>	35	2529183	2529534	<<<	351	0	>>>	cytochrome o ubiquinol oxidase subunit II	1.80E-202	R
AbsR-28	lysine-specific permease	>>>	36	3285829	3286033	>>>	204	136	>>>	hypothetical protein	7.50E-15	B
AbsR-29	hypothetical protein	>>>	14	3463191	3463452	>>>	261	169	>>>	putative vanillate O-demethylase oxygenase subunit	1.50E-12	T
AbsR-30	S-methylmethionine APC transporter	<<<	404	3623531	3623594	>>>	63	12	<<<	hypothetical protein	3.90E-09	T
AbsR-31	S-methylmethionine APC transporter	<<<	397	3623524	3623606	<<<	82	0	<<<	hypothetical protein	1.80E-158	T

Up ORF: Upstream Open Reading frame; dist Up ORF: distance of sRNA from Upstream Open Reading frame; UpORF dir: Upstream Open Reading frame direction; dir sRNA: direction of small RNA expression; len sRNA: length of sRNA; dist Dn ORF: distance of sRNA from downstream open reading frame.

### Experimental validation of predicted sRNA

**Figure 2:**
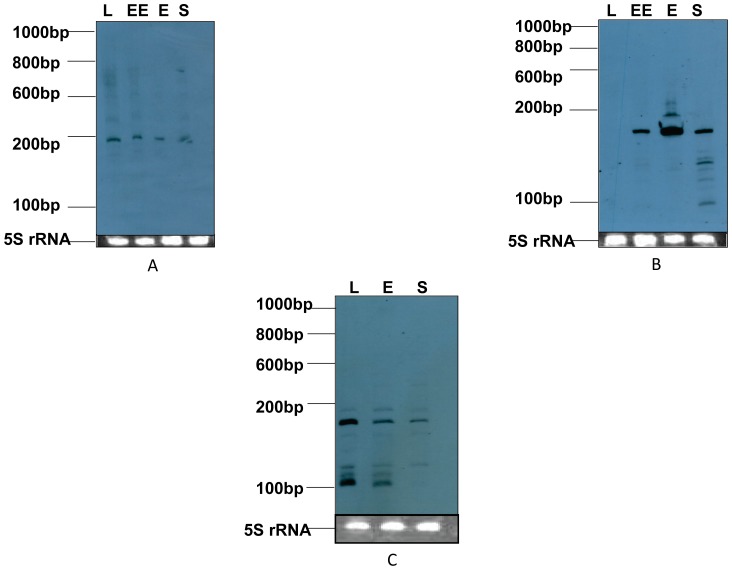
The expression of novel *A. baumannii* small RNA in different growth phases. Northern blot was used for determining the intracellular abundance of AbsRs over the course of growth (lag phase, L; early exponential phase, EE; exponential phase, E; stationary phase, S). Riborular low range RNA ladder (Fermentas, USA) sizes are shown to the left of the blot. Corresponding ethidium bromide stained 5S rRNA was used as loading control to ensure equal loading and shown below each Northern blot. (A) Northern blot of AbsR11, (B) Northern blot of AbsR25, (C) Northern blot of AbsR28.

The putative sRNAs predicted using sequence data of *A. baumannii* ATCC 17978 were verified in *A. baumannii* MTCC 1425 (ATCC 15308) which is an MDR strain. The expression of RNA species was examined in cells grown to lag, early exponential, exponential and stationary phase. The cells were also subjected to stress conditions, of which some are reminiscent of environmental conditions that *A. baumannii* cells encounter upon invasion of human wounds. These include temperature and osmotic stress. Out of 31 candidate genes, 10 candidate sRNA genes (*absR* 9, 11, 13, 14, 16, 17, 22, 24, 25, 28) were tested. These genes were chosen for validation as they were the most conserved (having lowest E values) among intergenic regions transcribing sRNA candidates of other sequenced *Acinetobacter* spp. and closely related bacteria as predicted by BLAST analysis. 78 nucleotides long specific riboprobes were designed for detection of signal in Northern blotting. For Northern blotting, total RNA of four different growth phases (lag, early exponential, exponential and stationary) was tested. Of the ten putative sRNA candidates analyzed, three (AbsR11, 25 and 28) showed positive signal on Northern blots (Figure 2A, B, C).

The Predicted length of AbsR11 sRNA was 351 nts (nucleotides) but in Northern blot a positive signal of 200 nts was obtained which was further confirmed by RACE mapping (Figure S1). AbsR11 sRNA was observed during all phases of bacterial growth with almost the same level of expression (Figure 2A).

AbsR28 was predicted to be 205 nts long and is located between A1S_2838 (encoding a lysine specific permease) and A1S_2839 (encoding a hypothetical protein),the experimentally proven length is approximately 180 nts (Figure S1). Its expression was also observed in all the three growth phases tested (Figure 2C).

AbsR25 sRNA showed a differential level of expression with no expression in lag phase, basal expression in early exponential and stationary phase and maximum expression in exponential phase (Figure 2B). The length of AbsR25 sRNA predicted by bioinformatic approaches is 333 nts and the location is between A1S_1321 and A1S_1322 genes, encoding putative hemolysin and dihydro-dipicolinate synthetase respectively. Northern blotting and RACE mapping showed that the transcript of AbsR25 sRNA was about 164 nts (Figure S1). The shortening in length might be due to processing of 3′ and 5′ ends of sRNA. Expression of AbsR25 was also tested under different conditions of temperature, osmotic and ethidium bromide stress.

**Figure 3:**
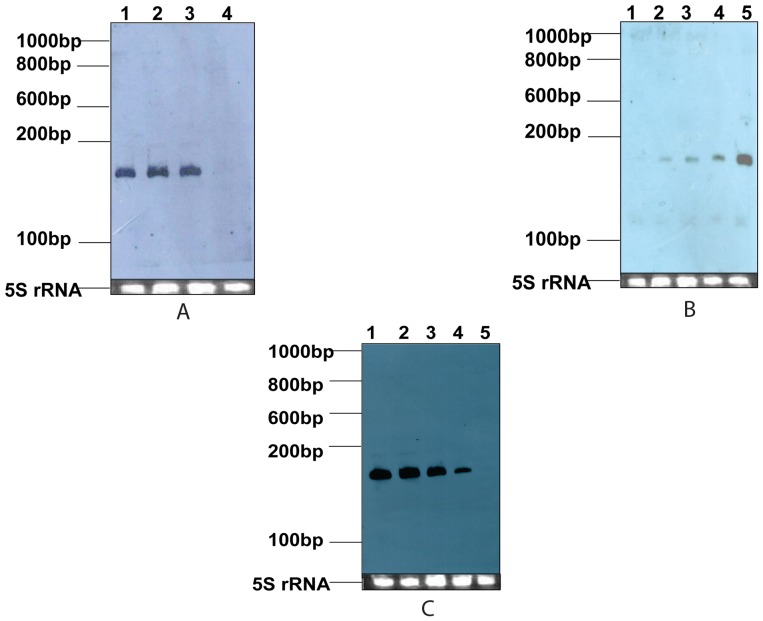
Northern blot showing expression of AbsR25 small RNA under different stress conditions. Northern blot showing the expression of AbsR25 small RNA candidate in Nutrient broth medium at different stress conditions is presented. (A) Total RNA was extracted from *A. baumannii* MTCC1425 cells growing at different temperature conditions (30°C, 37°C, 45°C, 50°C). 25 μg of total RNA sample of temperature stresses; 30°C, 37°C, 45°C, 50°C were loaded in lane 1 to 4 respectively. (B) Total RNA was extracted from *A. baumannii* MTCC1425 cells growing in nutrient broths containing 100, 200, 300, 400 and 500 mM of NaCl. 25 μg of extracted total RNA from above mentioned concentrations of NaCl were loaded from lane 1 to 5 respectively. (C) 25 μg of extracted total RNA of *A. baumannii* MTCC1425 cells growing in 0 μg/ml, 64 μg/ml, 128 μg/ml, 192 μg/ml and 256 μg/ml supplemented concentration of EtBr were loaded on PAGE from lane 1 to 5 respectively. Riborular low range RNA ladder (Fermentas, USA) sizes are shown to the left of the blot. Small RNA was detected by biotin labeled riboprobes designed to pair with the predicted small RNA. Corresponding ethidium bromide stained 5S rRNA was used as loading control to ensure equal loading.

For temperature stress, bacterial cultures were grown at 37°C, 45°C and 50°C for 30 minutes. The level of expression of AbsR25 sRNA was same at 30°C, 37°C and 45°C but no expression was observed at 50°C (Figure 3A).

Osmotic stress to *A. baumannii* cells was provided by growing cells under different concentrations of NaCl (100, 200, 300, 400 and 500 mM). As the concentration of NaCl increased, the expression of AbsR25 increased. This indicates that this sRNA may be necessary for survival of bacteria under osmotic stress conditions (Figure 3B).

For ethidium bromide (EtBr) stress, bacterial cells were grown in a gradient of EtBr concentration (0 μg/ml, 64 μg/ml, 128 μg/ml, 192 μg/ml and 256 μg/ml respectively). An increase in the concentration of EtBr decreased the level of expression of AbsR25 (Figure 3C). This indicates that this sRNA might have a role in the survival of the bacterium under EtBr stress. Since it is well documented in the literature that EtBr is a common substrate of many efflux pump families, the link between sRNA and efflux pumps was explored and the targets of this sRNA were predicted using bioinformatic approaches.

The expression of other predicted sRNAs was not observed in the tested conditions for the present study. These sRNA genes might be expressed in different growth and physiological conditions specific to the cellular needs.

### RACE mapping of validated sRNAs

Northern blots only provide information about the expression level and the approximate size of a transcript, but cannot detect the exact position of the 5′ and 3′ ends of sRNA. For precise detection of transcription start and stop site, rapid amplification of cDNA ends (RACE) analysis was performed. After RACE mapping the size of AbsR11, AbsR25 and AbsR28 were predicted to be 200 nts, 164 nts and 180 nts respectively (Figure S1). Sequences of sRNAs and its location in the genome are shown after BLAST analysis (Figure S1).

### Distribution of the identified sRNA genes in other bacteria

To determine whether the sRNA genes identified in this study are present in other microorganisms, the complementary DNA sequence of the sRNA genes was used to perform a BLAST search against the NCBI total sequence database (http://www.ncbi.nlm.nih.gov/Genebank/index.html). The results depicted in Table 3 show that none of the three identified sRNAs display sequence similarity with other bacterial species, indicating that these three sRNAs are novel. The result of AbsR11 sRNA BLAST showed that homologous sequences were found in all sequenced *Acinetobacter* species, indicating that AbsR11 may be *Acinetobacter* specific sRNA. Homologous gene sequences of *absR25* were found only in *A. baumannii* strains except SDF strain, indicating that AbsR25 could be species specific sRNA. In case of AbsR28, homologous sequences were found in all sequenced *Acinetobacter* species except *Acinetobacter* sp. ADP1 suggesting that this could also be *Acinetobacter* specific sRNA.

**Table 3:** Distribution and conservation of the identified sRNA gene sequence (*absR25*) in closely related bacteria.

Bacterial strains	AbsR11 (Identity out of 100% query coverage)	AbsR25 (Identity out of 100% query coverage)	AbsR28 (Identity out of 98% query coverage)
Aba 17978	100%	98%	98%
Aba AYE	99%	98%	99%
Aba ACICU	100%	98%	99%
Aba AB0057	99%	98%	99%
Aba AB307-0294	99%	98%	99%
Aba 1656-2	100%	98%	99%
Aba MDR-ZJ06	100%	98%	99%
Aba TCDC-AB0715	100%	98%	99%
Aba MDR-TJ	100%	98%	99%
Aba SDF	99%	N	98%
Aca PHEA-2	96%	N	89%
Aol DR1	95%	N	92%
A sp. ADP1	84%	N	N

Here in table; Aba, Acinetobacter baumannii; Aca, Acinetobacter calcoaceticus; Aol, Acinetobacter oleivorans; N, no similar sequence found.

### Prediction of AbsR25 sRNA target genes

*In silico* analysis was performed for prediction of AbsR25 target genes. For more specific results, two different programs (sRNATargetNB and IntaRNA) were used. sRNATargetNB uses Naïve Bayes probabilistic method for sRNA target gene prediction and takes secondary structure profile as the feature [59]. IntaRNA predicts interactions between two RNA molecules, and the scoring is based on the hybridization free energy and accessibility of the interaction sites in both molecules [60]. By using these two target prediction tools, 62 common putative target genes were predicted for AbsR25 (Table S5). The combined *in silico* data constitute the hypothetical regulon for AbsR25 and these genes were experimentally verified.

### Confirmation of AbsR25 target genes

**Figure 4:**
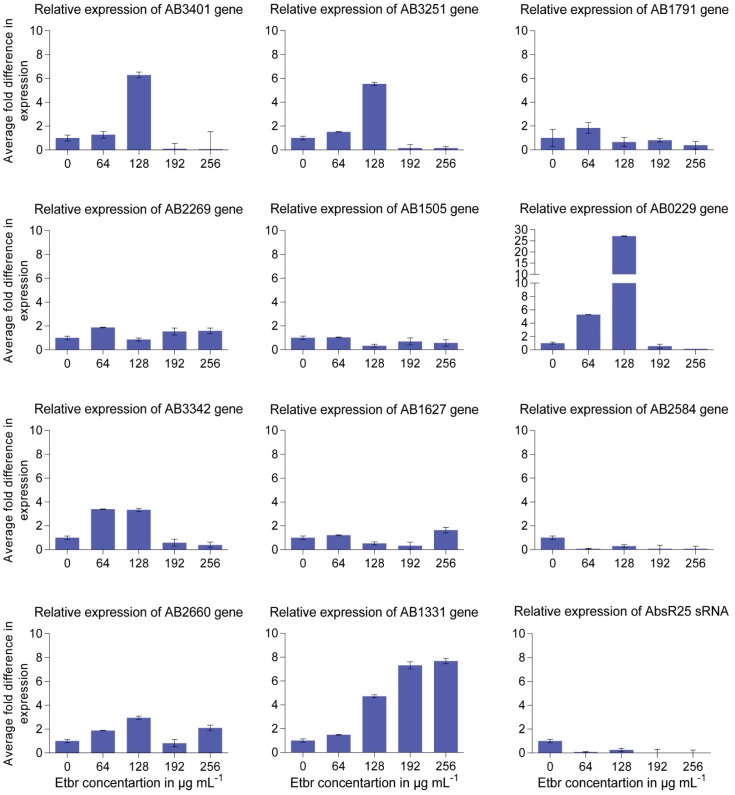
Real time expression of selected target genes. Relative expression of *in silico* predicted putative target genes [A1S_3401 (hypothetical protein), A1S_3251 (amino acid transporter LysE), A1S_1791 (putative tartrate symporter MFS superfamily protein), A1S_2269 (RtcR - Regulator of RNA terminal phosphate cyclase), A1S_1505 (hypothetical protein), A1S_0229 (hypothetical protein), A1S_3342 (putative arsenate reductase), A1S_1627 (hypothetical protein), A1S_2584 (major facilitator superfamily family drug transporter), A1S_2660 (RND efflux transporter), A1S_1331 (major facilitator superfamily transporter)] of AbsR25 sRNA under different concentrations of EtBr.

It is well characterized that expression of target genes is controlled by base pairing of sRNA with the cognate target mRNA. Base pairing of sRNA with its target mRNA not only affects translation but also leads to change in the stability of both sRNA and mRNA. To experimentally identify potential target genes of AbsR25 sRNA, the transcriptomes of *A. baumannii* were compared. As the levels of AbsR25 were found to decrease with increasing concentration of EtBr (in the earlier experiment described above, Figure 3C), the cells were grown in nutrient broth, in the presence of different concentrations of EtBr and in the absence of EtBr. For expression analysis of putative target genes, qRT-PCR was used. Out of 62 putative target genes, initially 11 genes were screened on the basis of having higher hybridization energy predicted by the IntaRNA program (Table 4). Out of these mRNAs, nine (AIS_0229, AIS_1331, AIS_3401, AIS_1627, AIS_2269, AIS_2660, AIS_3251, AIS_3342, AIS_1791) showed increased expression (Figure 4) of mRNA transcript relative to control (sample prepared without EtBr treated culture) (Table 4). The maximum change in expression (27.09 fold increase) was observed in case of AIS_0229 (hypothetical protein), 7.67 fold increase in expression in case of AIS_1331 (major facilitator superfamily transporter) and 6.2 fold increase in expression was observed in case of AIS_3401 (hypothetical protein) gene. Although the change in expression was observed in many genes, consistent increase in various concentrations of EtBr was observed only in case of AIS_1331 (major facilitator superfamily transporter gene) (Figure 4). The expression of AbsR25 sRNA was also tested in the same conditions which showed a decrease in its expression as the concentration of EtBr was increased from 0 μg/ml to 256 μg/ml. Conversely, as the concentration of EtBr was increased, the expression of AIS_1331 gene which encodes a major facilitator superfamily transporter or efflux pump increased at least six fold. This shows that AbsR25 negatively regulates the efflux pump transporter, AIS_1331 (major facilitator superfamily transporter) which controls the efflux of EtBr.

**Table 4:** Selected target genes for expression analysis by qRT-PCR.

S. No.	Putative target mRNA	Normalized relative expression of target mRNAs & AbsR25 in the presence of EtBr					Normalized relative expression of target genes in cells expressing AbsR25	
	**EtBr concentrations (μg/ml)**	**0**	**64**	**128**	**192**	**256**	**Uninduced**	**Induced**
1	A1S_3401 (hypothetical protein)	1	1.27	6.2	0.1	0.06	1	0.14
2	A1S_3251 (amino acid transporter LysE)	1	1.51	5.54	0.16	0.15	1	1.40
3	A1S_1791 (putative tartrate symporter MFS superfamily protein)	1	1.84	0.66	0.8	0.38	1	0.86
4	A1S_2269 (RtcR - Regulator of RNA terminal phosphate cyclase)	1	1.87	0.86	1.53	1.59	1	1.23
5	A1S_1505 (hypothetical protein)	1	1.04	0.32	0.69	0.56	1	1.23
6	A1S_0229 (hypothetical protein)	1	5.27	27.09	0.53	0.13	1	0.11
7	A1S_3342 (putative arsenate reductase)	1	3.38	3.34	0.58	0.39	1	0.19
8	A1S_1627 (hypothetical protein)	1	1.21	0.52	0.33	1.63	1	1.08
9	A1S_2584 (major facilitator superfamily family drug transporter)	1	0.06	0.29	0.07	0.06	1	1.12
10	A1S_2660 (RND efflux transporter)	1	1.87	2.95	0.81	2.09	1	0.51
11	A1S_1331 (major facilitator superfamily transporter)	1	1.49	4.72	7.31	7.67	1	0.42
12	AbsR25 sRNA	1	0.08	0.25	0.009	0.007	1	51.03

**Figure 5:**
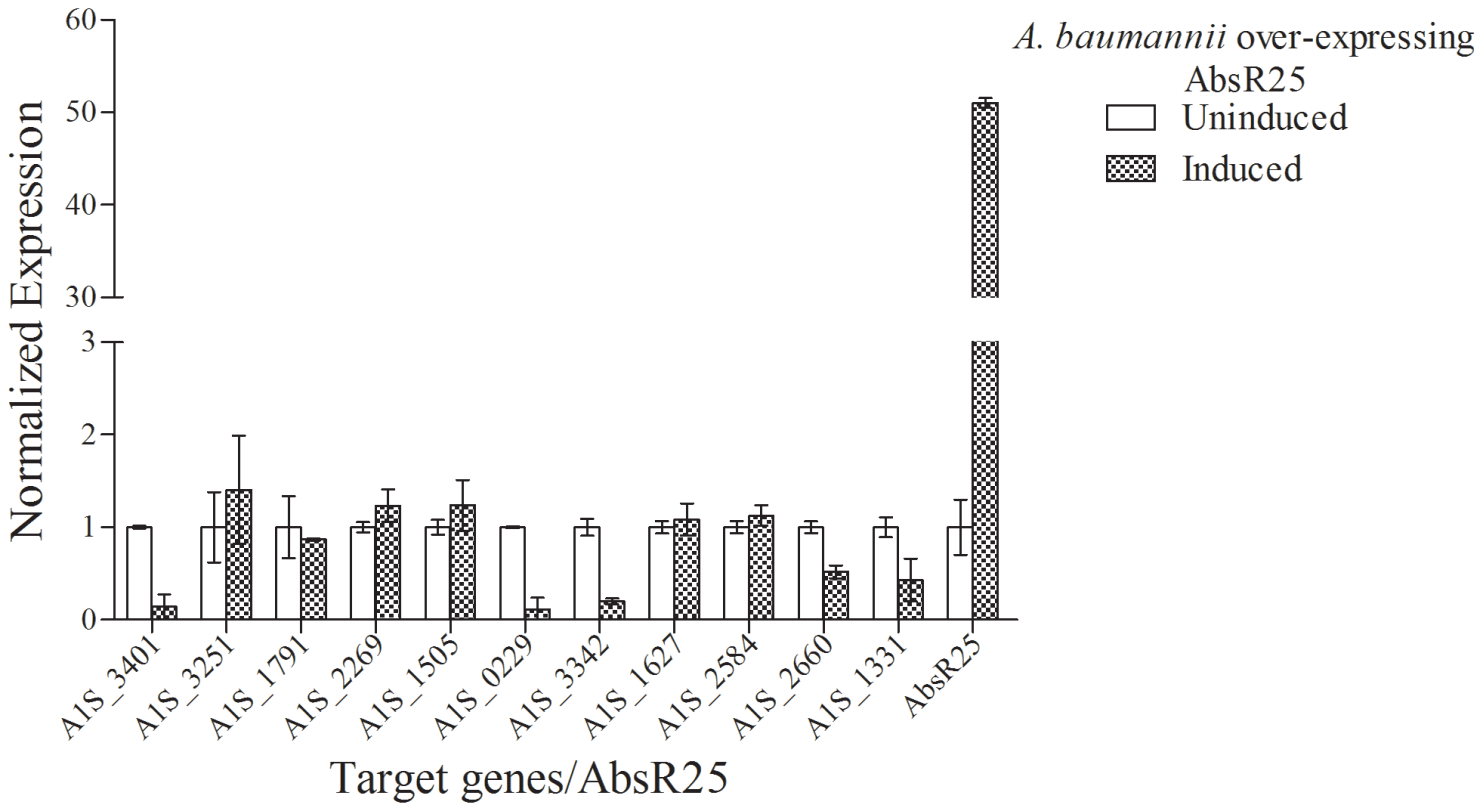
Effect of AbsR25 sRNA overexpression. Comparison of expression of predicted putative target genes in *A. baumannii* cells harbouring recombinant plasmid over-expressing AbsR25 sRNA. Expression of target genes was studied under arabinose induced and uninduced conditions. The experiments were performed in triplicates.

Since EtBr itself can affect the expression of some genes, therefore AbsR25 was over-expressed to determine bona-fide interactions with the target genes. *A. baumannii* strain designed to express higher amounts of AbsR25 was created by directional cloning of *absR25* gene in pPROBE-P_BAD_. Depending upon the cloning strategy, expression of *absR25* was controlled by P_BAD_ promoter (*A. baumannii* pPROBE-P_BAD_-*absR25*). The qRT-PCR analysis confirmed that over-expression of AbsR25 was successful in *A. baumannii* pPROBE-P_BAD_-*absR25* (Figure 5). No obvious difference was detectable in the growth behavior of *A. baumannii* pPROBE-P_BAD_-*absR25* under arabinose induced and uninduced conditions (data not shown). The expression of 11 putative target genes was also studied in *A. baumannii* pPROBE-P_BAD_-*absR25*. qRT-PCR was used for determination of expression profile of these genes. Out of these putative target genes, six (A1S_0229, A1S_1331, A1S_3401, A1S_2660, A1S_3342, A1S_1791) showed maximum repression in expression (Figure 5) of mRNA transcript relative to control (uninduced sample). The maximum repression in expression (9 fold) was observed in case of A1S_0229 (hypothetical protein), 7.14 fold repression in case of A1S_3401 (hypothetical protein) gene, 5 fold in case of 3342 (putative arsenate reductase) gene, 2.3 fold repression in case of A1S_1331 (major facilitator superfamily transporter) and 1.9 fold repression was observed in A1S_2660 (RND efflux transporter) gene. On the basis of similarity search (Table S6) it was found that out of six repressed genes; five had homologs belonging to different families of transporters. Of these five genes, three belong to the families of transporters which are most commonly implicated in drug transport. One of these genes (A1S_1331, major facilitator superfamily transporter) shows higher fold repression as compared to others which could be of significant value. For other genes (A1S_3251, A1S_2269, A1S_1505, A1S_1627, A1S_2584), a negligible increase in expression was detected.

## Discussion

In the present study we report the integration of computational and experimental methods to identify novel sRNAs in the nosocomial pathogen; *A. baumannii*. The bioinformatic prediction of sRNAs was based on screening the intergenic regions (IGR) of *A. baumannii* ATCC 17978 genome for sequence conservation, rho-independent termination and conserved secondary structures. As a measure to enhance the accuracy, two different query sets for prediction were prepared. First set contained the sequences from species closely related to *A. baumannii* (*P. aeruginosa*, *B. multivorans* and *S. maltophilia*) while the other set contained sequences from two *A. baumannii* strains (*A. baylyi* ADP1 and *Acinetobacter* spp. DR1). Prediction was carried out on both the sets and only the common results obtained from both the sets were considered for further analysis. Two web based programs (sRNAPredict and QRNA) were used for increasing the sensitivity of the sRNA prediction. The sRNAPredict algorithm is based on two types of information, first the location of transcriptional signal and second primary sequence conservation of IGRs. On the other hand QRNA algorithm is based on conservation of secondary structures of RNA for prediction. As a result of this exercise a list of 31 putative sRNA molecules was generated which satisfied the set conditions and contained common sequences predicted as sRNA from both the groups.

On the basis of sequence conservation and lower E-value in BLAST analysis, 10 out of the 31 putative sRNA molecules were selected for experimental validation. Northern blotting was performed to detect the expression of the selected 10 putative sRNAs in *A. baumannii* MTCC 1425 (ATCC 15308). The MTCC 1425 strain is an MDR strain showing resistance to 12 antibiotics belonging to different chemical classes (data not shown). Positive signal on Northern blot was detected for three putative sRNA species namely; AbsR11, AbsR25 and AbsR28. The sizes of all the three molecules were shorter than the predicted size which might be due to processing at the 5′- or the 3′- end. This size difference was confirmed by RACE mapping. The size of AbsR11, AbsR25 and AbsR28 was found to be 200 nts, 164 nts and 180 nts, respectively corresponding to the sizes predicted by Northern blot. BLAST analysis revealed that AbsR11 and AbsR28 are conserved among the *Acinetobacter* spp. but no such homology existed in other genera, making them genus specific. However, AbsR25 was found conserved only among *A. baumannii* strains making this sRNA species specific (Table 3). Since no similarity of these sRNAs with already identified sRNAs was found in other bacteria, these sRNAs are novel in nature.

The expression of AbsR11 and AbsR28 was independent of the stage of growth of *A. baumannii* as the Northern blot using RNA from different growth phases (lag, early exponential, exponential and stationary) showed almost equal level of expression. However, in case of AbsR25, the expression of the said sRNA was not detectable in lag phase, basal in early exponential and stationary phase and maximum in exponential phase. This intriguing observation about AbsR25 led to further investigation on the molecule. The differential expression may indicate its role in stress conditions when there is a scarcity of nutrients. To establish the role of AbsR25 in stress response, its expression profile was studied under gradients of different stress conditions like osmotic, temperature and EtBr stress. These conditions represent the stress that the pathogen is likely to encounter when it makes entry into the host. No change in the expression of AbsR25 was observed in the temperature range of 30°C to 45°C. At 50°C expression of AbsR25 could not be detected which may be due to the difficulty of the bacterial strain to grow (Figure 3A). In contrast, the expression of AbsR25 varied with the concentration of salt (NaCl) in the medium. An increase in the expression of AbsR25 was seen with progressively increasing concentrations of NaCl. This further strengthens the hypothesis that the small RNA may be involved in the cellular mechanism to cope up with environmental stress.

A significant observation was made in the case of EtBr stress. The expression of AbsR25 was found inversely related to the concentration of EtBr in the growth medium of *A. baumannii* (Figure 3C). This observation suggests that AbsR25 might play an important role in efflux pump regulation and drug resistance. Interestingly, most of the predicted targets for AbsR25 were efflux pumps with high hybridization energy. This indicates that AbsR25 might be regulating, directly or indirectly, the expression of efflux pumps. To validate this hypothesis, qRT-PCR was performed with the cDNA of the EtBr stressed culture. Out of the mRNA target genes tested, maximum variation in expression was shown by AIS_0229 (hypothetical protein), AIS_1331 (major facilitator superfamily transporter), AIS_3401 (hypothetical protein) and AIS_3251 (amino acid transporter LysE). Further experiments will be needed to verify these targets. qRT-PCR results reveal that the presence of EtBr in culture medium of *A. baumannii* results in decreased AbsR25 expression level and upregulated A1S_1331 mRNA levels which specifies an MFS pump in *A. baumannii*. It is evident that the expression of mRNA specifying AIS_1331 transporter increased uniformly with the increase in concentration of EtBr which also coincided with a steady decrease in the expression of AbsR25 sRNA. It is proposed that this sRNA might be involved in negative regulation of the AIS_1331 transporter. In the absence of EtBr in the culture, sRNA expression was found high enough to prevent the expression of the transporter, however, when the concentration of EtBr increased, the expression of sRNA decreased and the transporter is available for fluxing out the EtBr for survival of the bacterium.

However, EtBr is known to affect the expression of a lot of genes. Therefore, to overcome the effect of EtBr itself on the expression of said genes, AbsR25 was overexpressed in *A. baumannii* and the effect of its overexpression was studied on target genes. The change in expression of the target genes was in accordance with the observations made earlier (Table 4). The increased expression of AbsR25 resulted in 9, 7.14, 5, 2.3, 1.9 and 1.1 fold repression of A1S_0229, A1S_3401, A1S_3342, A1S_1331, A1S_2660, A1S_1791 genes respectively. As is evident from the experiment, the affected genes include five putative transporters belonging to different families. Out of these putative transporters, AIS_1331 has been confirmed as an MFS transporter in a separate study in our group (unpublished results). On the basis of these observations, AbsR25 could be implicated in negative regulation of the expression of A1S_1331 transporter gene, which is in close agreement with EtBr treatment of *A. baumannii* cells. The repression of the genes indicates that the regulation of A1S_1331 transporter gene by AbsR25 sRNA is achieved at the mRNA level.Putative pairings between AbsR25 and AIS_1331mRNA have been identified but require direct experimental support. AbsR25 might also regulate other targets encoded at separate locations on the chromosome. Since it is well established that efflux pumps regulate clinically relevant resistance to antibiotics [65], these findings provide a new perspective for future research into the mechanisms responsible for antibiotic resistance.

The identification, experimental confirmation, and preliminary investigation of novel and distinctive sRNAs from the nosocomial pathogen *A. baumannii* contribute to the ongoing attempt to characterize and comprehend the function of these significant RNA molecules. This is the first report of novel sRNAs in *A. baumannii* and preliminary result of involvement of a sRNA, AbsR25, in regulating an efflux pump in clinically significant MDR strain. Further experiments are needed to disclose elucidations to the exciting observation of the involvement of AbsR25 sRNA in regulation of expression of efflux pumps and hence in multiple drug resistance.

## Supporting Information

Figure S1 **Sequences of sRNAs and its location in the genome after BLAST analysis.**
(DOC)

Table S1 **QRNA output for first study.**
(XLS)

Table S2 **Output of sRNAPredict for first study.**
(XLSX)

Table S3 **QRNA output file for second study.**
(XLSX)

Table S4 **sRNAPredict output for second study.**
(XLSX)

Table S5 **Putative target genes of AbsR25 predicted by bioinformatic analysis.**
(XLSX)

Table S6 **BLAST analysis of six repressed target genes.**
(DOC)
