# Supplementary material for: Identification of Novel Regulatory Small RNAs in Acinetobacter baumannii
Source: PLoS One. 2014 Apr 4;9(4):e93833. doi: 10.1371/journal.pone.0093833 (PMC3976366; doi:10.1371/journal.pone.0093833)
Supplement: Figure S1 — Sequences of sRNAs and its location in the genome after BLAST analysis. (DOC) [file pone.0093833.s001.doc]

**AbsR11** >TCAGACGACTTTATGTGTAGCTTAGTTTGTCGATATGAGGCATAATGCCCTACAACGTAGCGGTGTCACATCATTGAGAAGTCTAATTTTTAAGACTAGCGTTCTCAATCATGCGACACTTTAATCGCTATCCCATATTTAATTAGGGAATGGGACTCTTCACCGAGGTTGTAAAATGAGACAAACGATTTTAGCTGTAT


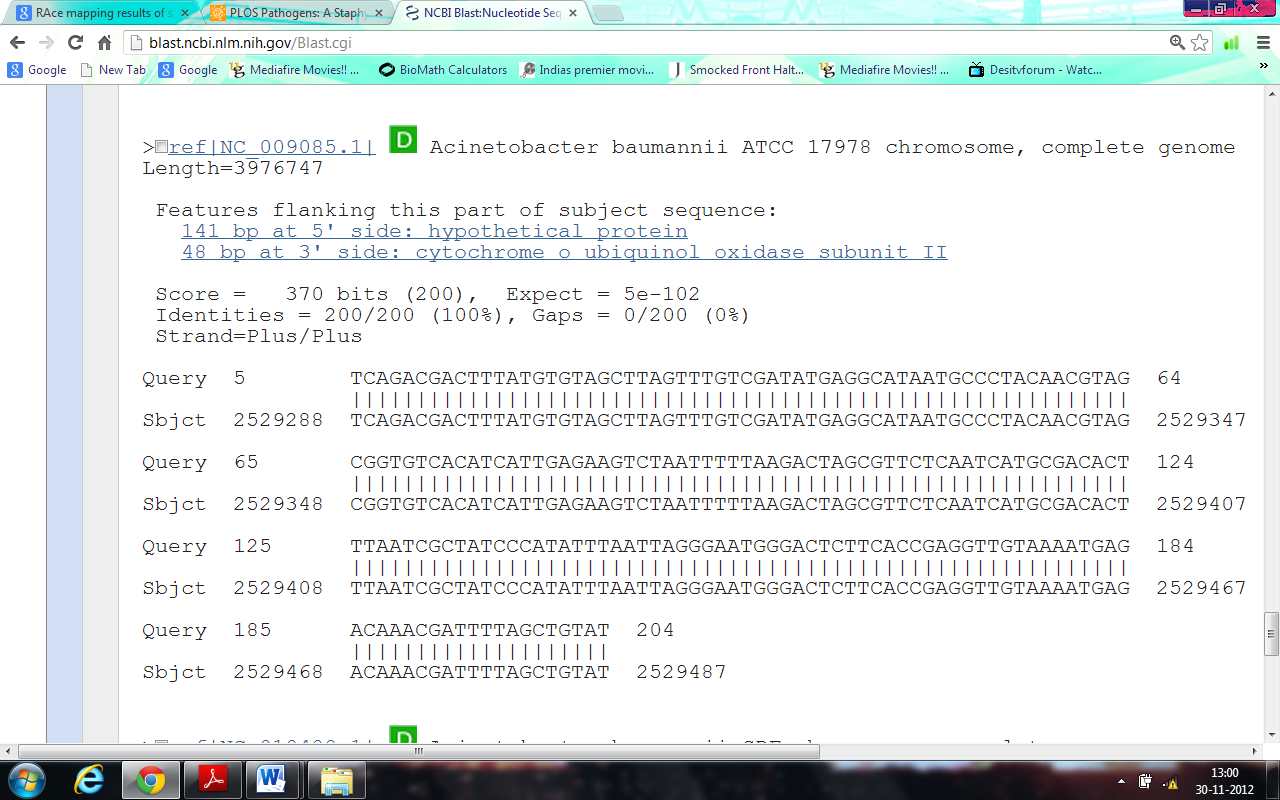


**AbsR25**

**>**CCTTTTAAATCATGTGTAGGACCGAACTATGATGCAAGCAATGTTTTTCTTTAGTGCTGGAGCATTTTTTGGTTATTGCTATCGTCATTACGATGCTAAAGTTAAAAAAGCCAATGATTCAAAATAAATGATTAAAAAAGCCTGCTTCTTGAGTAGGCTTTTTT


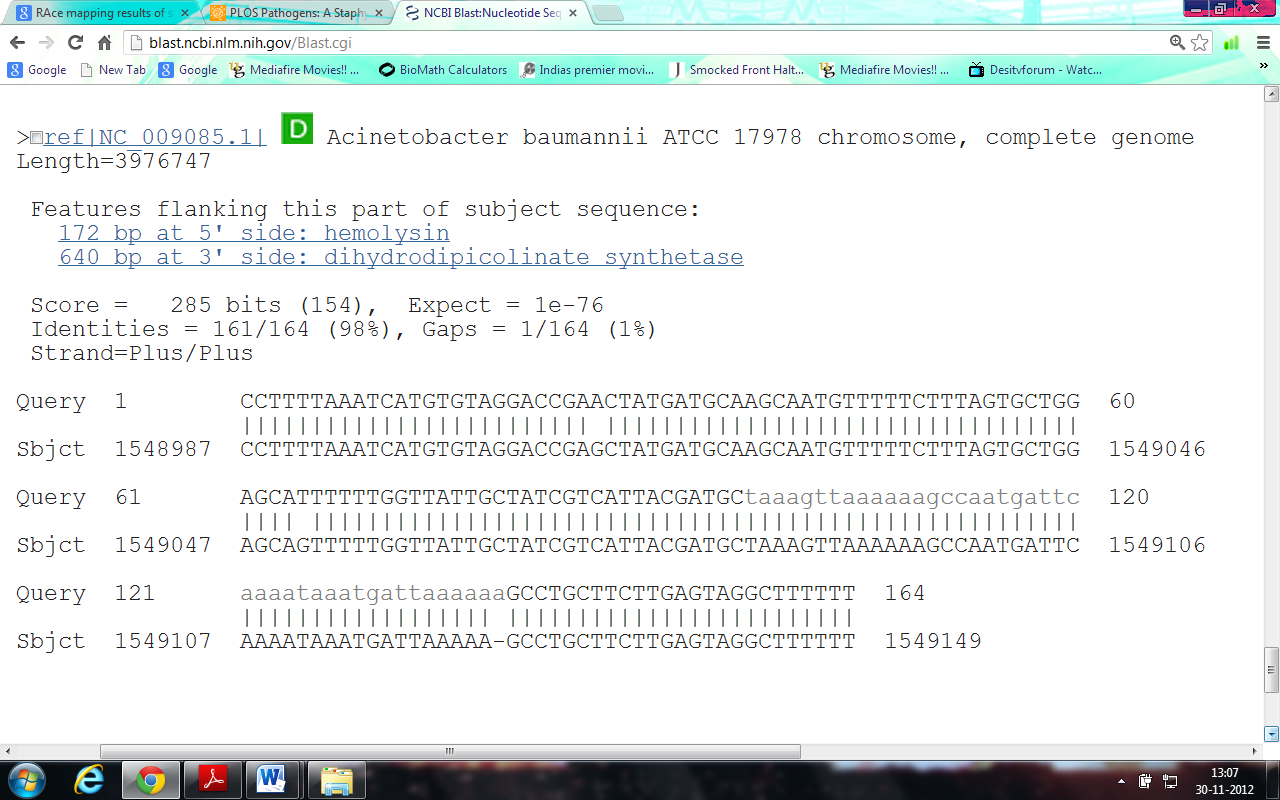


**AbsR28**

**>**ATATTTTCAACGGCACTTTTTAAAAAAGGAGGACATCATGCCAACACTACAAGGTAAAACACAGTAACGATTTGGCACGATGTCAGCTCACGGACAAGTGTACAAATTTAATTCACTTGTCCGAATGCTTGAATTTAAGACCTAGCATTCGCTAGGTTTTTTTATTCCTATTCGGACAAT


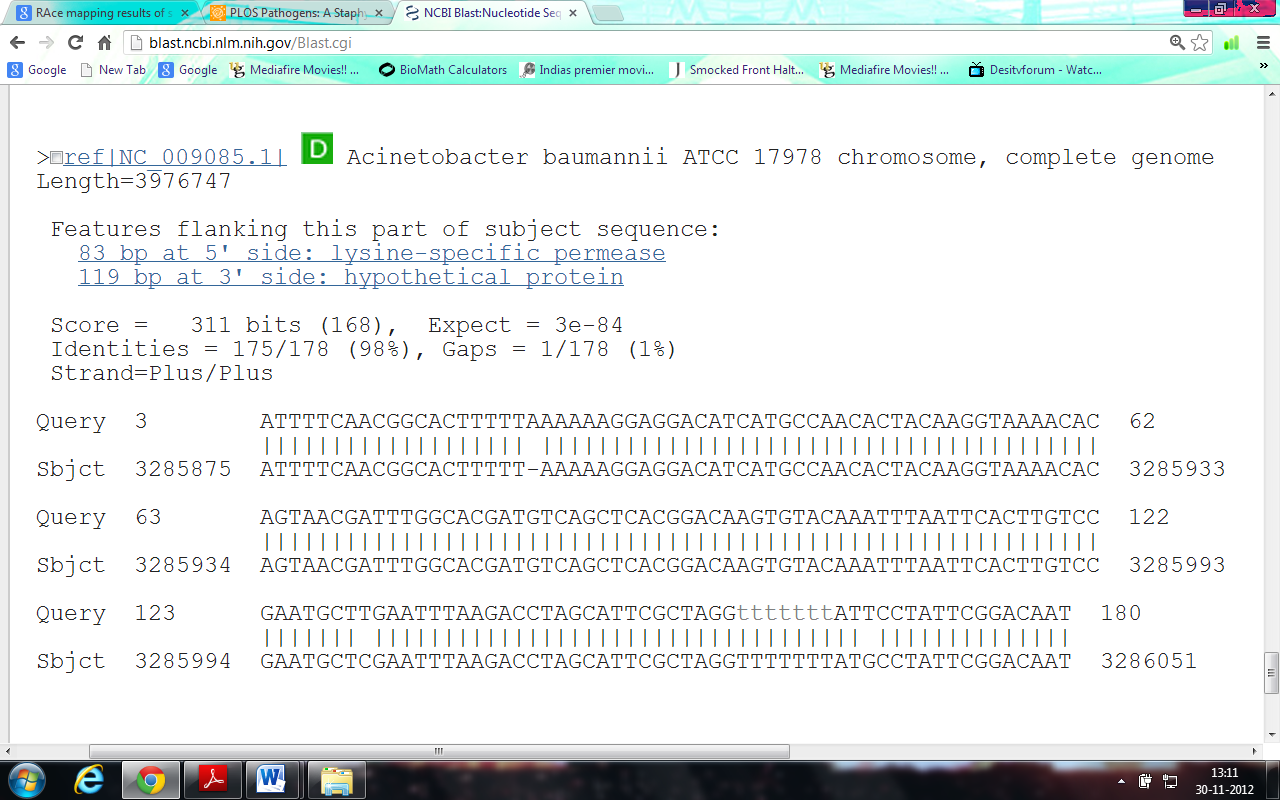


**Supplementary data figure 1.** Shows sequence of AbsR11, 25 & 28 and its alignment with *Acinetobacter baumannii* ATCC17978 genome
