# Supplementary material for: Identification of Novel Regulatory Small RNAs in Acinetobacter baumannii
Source: PLoS One. 2014 Apr 4;9(4):e93833. doi: 10.1371/journal.pone.0093833 (PMC3976366; doi:10.1371/journal.pone.0093833)
Supplement: Table S6 — BLAST analysis of six repressed target genes. (DOC) [file pone.0093833.s007.doc]

| **S. No.** | **Gene name** | **Organism name** | **Identical gene** | **Query coverage (%)** | **Identity (%)** |
| --- | --- | --- | --- | --- | --- |
| 1 | A1S_0229 | *Enhydrobacter aerosaccus* | ABC transporter permease | 98 | 37 |
| 2 | A1S_3401 | *Xanthomonas campestris* | Lipoprotein | 81 | 42 |
| 3 | A1S_3342 | *Marinomonas* sp.M47T1 | Arsenate Reductase like protein | 95 | 51 |
| 4 | A1S_1331 | *Bacillus cereus* | MFS transporter permease | 99 | 86 |
| 5 | A1S_2660 | *Xanthomonas albilineans* | RND superfamily protein | 99 | 55 |
| 6 | A1S_1791 | *Pseudomonas* sp.M47T1 | MFS transporter | 94 | 68 |

**Table S6. BLAST analysis of six repressed target genes**
